# Machine learning forecasting for COVID-19 pandemic-associated effects on paediatric respiratory infections

**DOI:** 10.1136/archdischild-2022-323822

**Published:** 2022-08-10

**Authors:** Stuart A Bowyer, William A Bryant, Daniel Key, John Booth, Lydia Briggs, Anastassia Spiridou, Mario Cortina-Borja, Gwyneth Davies, Andrew M Taylor, Neil J Sebire

**Affiliations:** 1 Great Ormond Street Hospital for Children, London, UK; 2 NIHR GOSH Biomedical Research Centre, London, UK; 3 Population, Policy and Practice Research and Teaching Department, UCL Great Ormond Street Institute of Child Health, London, UK; 4 UCL Institute of Cardiovascular Science, London, UK

**Keywords:** respiratory, information technology

## Abstract

**Objective:**

The COVID-19 pandemic and subsequent government restrictions have had a major impact on healthcare services and disease transmission, particularly those associated with acute respiratory infection. This study examined non-identifiable routine electronic patient record data from a specialist children’s hospital in England, UK, examining the effect of pandemic mitigation measures on seasonal respiratory infection rates compared with forecasts based on open-source, transferable machine learning models.

**Methods:**

We performed a retrospective longitudinal study of respiratory disorder diagnoses between January 2010 and February 2022. All diagnoses were extracted from routine healthcare activity data and diagnosis rates were calculated for several diagnosis groups. To study changes in diagnoses, seasonal forecast models were fit to prerestriction period data and extrapolated.

**Results:**

Based on 144 704 diagnoses from 31 002 patients, all but two diagnosis groups saw a marked reduction in diagnosis rates during restrictions. We observed 91%, 89%, 72% and 63% reductions in peak diagnoses of ‘respiratory syncytial virus’, ‘influenza’, ‘acute nasopharyngitis’ and ‘acute bronchiolitis’, respectively. The machine learning predictive model calculated that total diagnoses were reduced by up to 73% (*z*-score: −26) versus expected during restrictions and increased by up to 27% (*z*-score: 8) postrestrictions.

**Conclusions:**

We demonstrate the association between COVID-19 related restrictions and significant reductions in paediatric seasonal respiratory infections. Moreover, while many infection rates have returned to expected levels postrestrictions, others remain supressed or followed atypical winter trends. This study further demonstrates the applicability and efficacy of routine electronic record data and cross-domain time-series forecasting to model, monitor, analyse and address clinically important issues.

## Abstract (precis)

Retrospective study looking at Machine Learning Forecasting for Covid 19 Pandemic Associated effects on paediatric respiratory viruses.

**What is already known on this topic?**

- The literature states that (non-COVID-19) respiratory diagnoses have broadly reduced during the periods of government interventions as resulting from the COVID-19 pandemic, across the world.
- General reductions in respiratory infection diagnoses are generally in contravention with the typical seasonal trends.
- Research has predicted an increase in respiratory infections once government interventions and restrictions are removed.

**What this study adds?**

- This study analyses respiratory infections observed at a specialist children’s hospital during and after the implementation of restrictions resulting from the COVID-19 pandemic.
- The results show a significant reduction in rates of major respiratory diagnoses during restrictions but further illustrate the variation in responses post-restrictions.
- The study demonstrates how open-source, cross-domain, forecasting tools can be applied to routine health record activity data to provide evaluation of deviations from historical trends.

**How this study might affect research, practice or policy**

- This study shows that, in our population, hypothesised excess post-COVID-19 respiratory syncytial virus infections did not occur, with implications for health policy planning.
- The results indicate that rates for several respiratory infections continue to remain below typical pre-COVID-19 levels, and further research is required to model future effects.
- The electronic health record data-based forecasting method, using cross-domain tools, is applicable to a range of health policy applications, including service usage planning and case surge detection.

## Introduction

The COVID-19 pandemic had a major impact on healthcare services, with significantly reduced service utilisation.^1^ In addition, the mitigation measures implemented, such as lockdowns, social distancing and personal protective/hygiene actions, have significantly reduced rates of other infectious agents, for example, transmission of norovirus.^2^ Previous pandemics, such as influenza, have demonstrated that associated public health measures can impact rates of other respiratory infections such as respiratory syncytial virus (RSV),^3^ and reduced rates of RSV infection and other respiratory pathogens have been reported in several countries during the COVID-19 pandemic.^4–8^

The value of routine electronic health record (EHR) data for research is increasingly recognised and has been highlighted by the pandemic,^9–11^ and the UK Government has recently published a data strategy emphasising the value of healthcare data for secondary purposes.^12^ The aim of this study is to analyse routine electronic patient record data from a specialist children’s hospital to examine the effect of the COVID-19 pandemic mitigation measures on rates of seasonal respiratory infections compared with expected rates using an openly available transferable machine learning model.

## Methods

### Design

We performed a retrospective longitudinal study of coded respiratory disorder diagnoses made at the Great Ormond Street Hospital for Children (GOSH), a specialist paediatric hospital in London, that typically receives 280 000 patient visits per year and includes several large paediatric intensive care units.

The respiratory disorder data were extracted and aggregated from the Epic patient-level EHR and legacy clinical data warehouses^13^ using a bespoke Digital Research Environment.^14^ Diagnoses were labelled with codes from the International Statistical Classification of Diseases and Related Health Problems 10th Revision (ICD-10).^15^ All diagnoses from inpatients and outpatients recorded between 1 January 2010 and 28 February 2022 were collected for the study.

The diagnosis rates and trends of four respiratory disease categories that are reported to be particularly prevalent during the UK winter were analysed in this study (‘Respiratory Infection due to the Respiratory Syncytial Virus’ (RSV), ‘Respiratory Infection due to the Influenza Virus’, ‘Acute Nasopharyngitis due to any Virus’ and ‘Acute Bronchiolitis due to any Virus (excluding RSV)’). In addition, diagnoses were aggregated into categories based on respiratory hierarchical groupings of ICD-10 to provide a wider picture of diagnosis rates and seasonal trends^15^ (the full list of associated ICD-10 codes for each aggregated category is shown in online supplemental table 1).

10.1136/archdischild-2022-323822.supp1 Supplementary data

Each diagnosis category was divided into three time periods, corresponding to before, during and after the enforcement of national restrictions in England in response to the COVID-19 pandemic. The prerestriction period was designated as 1 January 2010–25 March 2020. The during restriction period was designated from 26 March 2020 (the date ‘The Health Protection (Coronavirus, Restrictions) (England) Regulations’ legally came into force) to 18 July 2021. The postrestriction period was taken from 19 July 2021 (the date ‘The Health Protection (Coronavirus, Restrictions) (Steps etc.) (England)’ was revoked) to 28 February 2022.^16^ England was subject to a range of interventions in the period during restrictions. At their most stringent, these restrictions included full national ‘lockdowns’ where meeting was disallowed, and it was a legal offence to leave your place of living except for a small range of ‘essential activities’. Conversely, at their least stringent, the restrictions permitted gatherings of up to 30 people and only had requirements for face coverings in enclosed spaces and minor personal social distancing measures.

All analysis and modelling for this study were carried out using the R programming language.^17^

All data were deidentified using the established digital research environment mechanisms, with analysis carried out in a secure virtual environment; no data left the hospital during the study.

### Statistical analysis

For each respiratory disorder diagnosis category, data for the cohort of patients with an associated ICD-10 diagnosis were extracted, and the start date of the period of diagnosis was identified. The daily diagnosis frequency (diagnoses/day) was calculated for each diagnosis category by aggregating the diagnosis dates of all patients with a diagnosis in the category across the period.

The diagnosis rate data were sparse for some categories; therefore, a 30-day moving average filter^18^ with a centre-aligned, rectangular window was applied to the raw diagnosis frequency series to provide an averaged representation of the diagnosis rate trends, y(t) , that was used for the subsequent analysis and modelling.

### Statistical modelling

To understand the impact of restrictions on GOSH diagnosis rates for each category, a statistical model for the typical trend was built from the diagnosis rate trends for the prerestrictions period using the Prophet forecasting procedure.^19^ Prophet is a robust, open source tool that fits additive and multiplicative seasonal models to time-series data that have strong cyclical/seasonal effects. With Prophet, an input time-series is decomposed into a non-periodic trend that changes non-linearly over time, multiple periodic seasonalities, an irregular holiday effect and a noise signal. Prophet fits the model to the input time-series within the Bayesian statistical inference framework with Markov chain Monte Carlo (MCMC) sampling implemented in the Stan programming language.^19^

For this study, the diagnosis rate model was designed as a multiplicative model, as follows.

$$y\left(t\right)=g\left(t\right)\cdot s\left(t\right)\cdot \epsilon_{t}$$

where $y\left(t\right)$ is the diagnosis rate time series, $g\left(t\right)$ is the non-periodic trend modelled as a piecewise linear trend with changepoints, $s\left(t\right)$ is the annual periodic seasonal trend modelled as a five term Fourier series, and $\epsilon_{t}∼N\left(0,\sigma^{2}\right)$ is a normally distributed model error function. A multiplicative model, whereby the trends and seasonalities are multiplied together to model the time-series, was used because diagnosis rates clearly showed annual seasonality to be approximately proportional to the overall trend. Details of the implementation of $g\left(t\right)$ and s(t) are available elsewhere.^19^

Since $y\left(t\right)\geq 0$ the multiplicative model was log-transformed and implemented as the following additive model

$$log_{\Delta }\;\left(y\left(t\right)\right)=log_{\Delta }\;\left(g\left(t\right)\right)+\log_{\Delta }\;\left(s\left(t\right)\right)+\epsilon_{t}$$

where x is the input diagnosis rate, $log_{\Delta }\;\left(x\right)=log\left(x+\Delta \right)$ approximates the log transformation and is finite for zero valued x for an arbitrary small constant ∆ .

To quantify the degree of seasonality in each diagnosis category, a ‘Seasonality Amplitude’ score was calculated from the Prophet model generated for each diagnosis category. The score, $a_{score}$ , was calculated as the ratio of the peak-to-peak amplitude, $a_{peak-to-peak}$ , and the peak amplitude, $a_{peak}$ , of the model forecast for the year immediately prior to the introduction of restrictions.

$$a_{score}=\frac{a_{peak-to-peak}}{a_{peak}}$$

To understand the significance of any deviation in the observed diagnosis rate from that predicted by the Prophet models, discrete daily *z*-scores were calculated, as follows:

$$Z_{i}=Z_{q}\left(P\left[{\hat{Y}}_{i}\leq Y_{i}\right]\right)$$

where $z_{i}$ is the i -th observed diagnosis rate *z*-score, $y_{i}$ is the i -th observed diagnosis rate, ${\hat{Y}}_{i}$ is the random variable defining the i -th value of the posterior predictive distribution from the raw MCMC samples in Prophet and $Z_{q}(.)$ is the mapping of probability quantiles to *z*-scores.

## Results

Data from 30 199 patients with a diagnosis from Chapter X ‘Diseases of the respiratory system’ of ICD-10 at the centre between 1 January 2010 and 28 February 2022 were included in the study, with a total of 141 003 diagnosis records in the dataset (including repeats). Full summary statistics for the study population are shown in table 1.

**Table 1:** Table of summary characteristics for the population of diagnoses analysed in the study

Characteristic	Value ( n =106 222) (%)
Sex	
Female	42.30
Male	57.69
Ethnic groups	
White	42.66
Not stated	23.30
Asian or Asian British	13.36
Black, black British, Caribbean or African	8.80
Other ethnic group	7.77
Mixed or multiple ethnic groups	4.11
Age at diagnosis	
Minimum	0.00 years
Quartile 1	1.39 years
Median	4.20 years
Quartile 3	9.48 years
Maximum	18.00 years

A total of 1060 diagnoses of ‘RSV’, 471 diagnoses of ‘Influenza’, 2214 diagnoses of ‘Acute Nasopharyngitis’ and 1568 diagnoses of ‘Acute Bronchiolitis (excl. RSV)’ were made across the period of study. Online supplemental table 1 shows the patient cohort summary for these diagnosis categories during the three time periods, in addition to those from the ICD-10 hierarchy.

**Figure 1:**
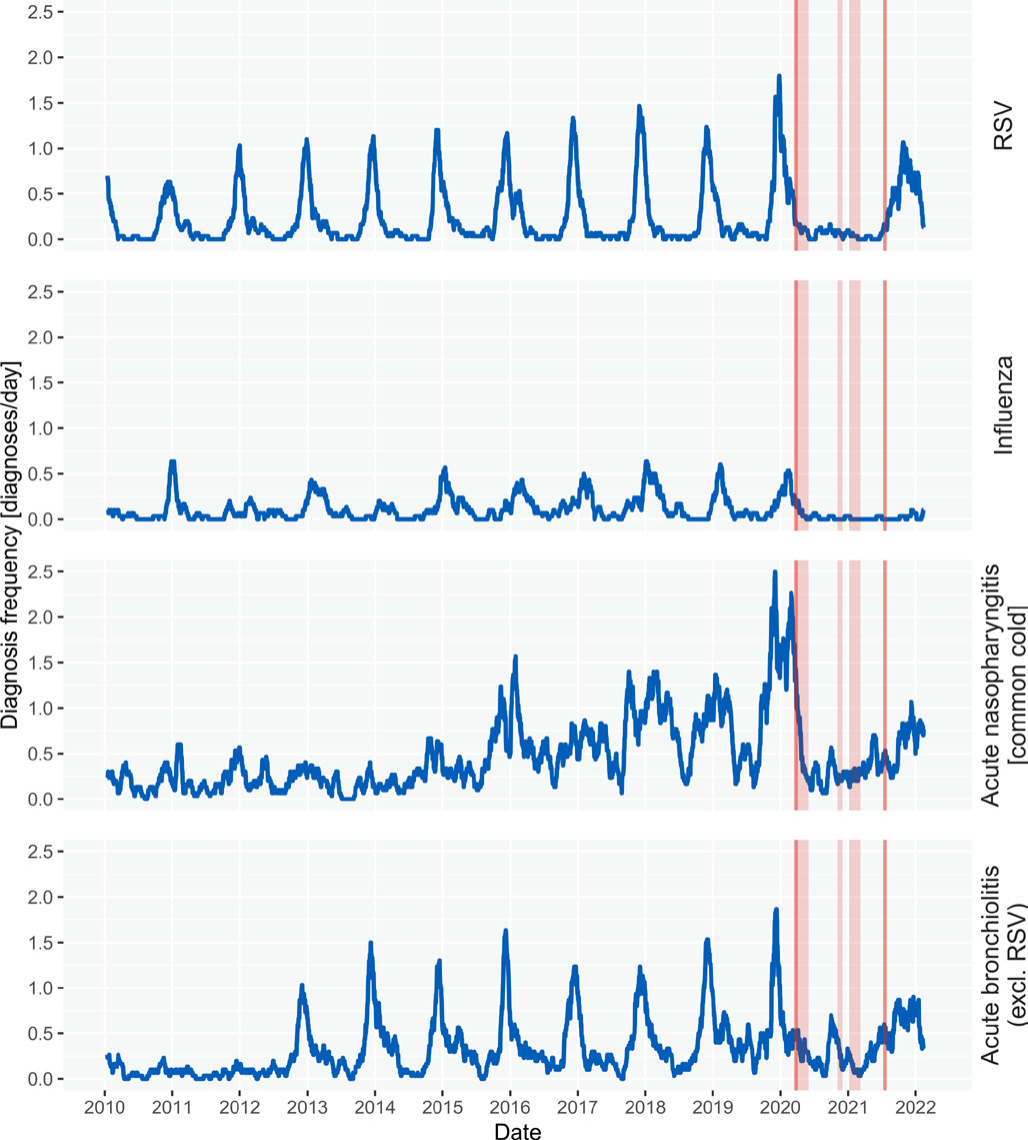
Diagnosis frequency plots for the four commonly seasonal respiratory disease categories. The blue line shows the observed 30-day moving average of daily diagnosis rate between 2010 and 2022. The vertical dark red lines define the start and end of widespread restrictions in response to the COVID-19 pandemic in England, UK. The light red sections show the three periods of national lockdowns.

The 30-day moving average diagnosis rates for the respiratory disorder diagnosis categories are shown in figure 1. The four diagnosis rate plots for the respiratory disorder diagnosis categories show clear seasonal trends and exhibit peaks in winter months and troughs in summer months.

For RSV, the prerestrictions period maximum diagnosis frequencies were 1.8 diagnoses/day. During the restrictions period, the maximum was 0.17 diagnoses/day, representing an 91% reduction. These results are shown for the other categories in table 2.

**Table 2:** Table of peak diagnosis rate values for the respiratory disease categories across the three time periods: prerestrictions, during restrictions and postrestrictions

Diagnosis	ICD-10 codes	Prerestrictions (1 January 2010–25 March 2020)		During restrictions (26 March 2020–18 July 2021)			Postrestrictions (19 July 2021–28 February 2022)		
		Peak diagnosis rate(diagnoses/day)	Date of peak	Peak diagnosis rate(diagnoses/day)	Date of peak	Change relative to prerestrictions (diagnoses/day) (%)	Peak diagnosis rate (diagnoses/day)	Date of peak	Change relative to prerestrictions (diagnoses/day) (%)
**Seasonal respiratory infection diagnosis categories**									
RSV	B974, J121, J205, J210	1.80	25 December 2019	0.17	22 September 2020	−1.63 (−90.74)	1.07	24 October 2021	−0.73 (-40.74 %)
Influenza	J09, J10, J11	0.63	28 December 2010	0.07	28 June 2020	−0.57 (−89.47)	0.10	08 December 2021	−0.53 (-84.21 %)
Acute nasopharyngitis (common cold)	J00	2.50	3 December 2019	0.70	18 May 2021	−1.8 (−72)	1.07	09 December 2021	−1.43 (-57.33 %)
Acute bronchiolitis (excl. RSV)	J211, J218, J219	1.87	9 December 2019	0.70	1 October 2020	−1.17 (−62.5)	0.90	21 December 2021	−0.97 (-51.79 %)
**ICD-10 hierarchy respiratory categories**									
Diseases of the respiratory system	J00-J99	23.93	28 February 2020	21.33	12 July 2021	−2.6 (−10.86)	23.93	24 January 2022	0 (0 %)
Acute upper respiratory infections	J00-J06	4.03	28 February 2020	1.63	12 July 2021	−2.4 (−59.5)	2.43	09 February 2022	−1.6 (-39.67 %)
Influenza and pneumonia	J09-J18	2.70	20 February 2020	2.03	15 April 2021	−0.67 (−24.69)	2.80	18 January 2022	0.1 (3.7 %)
Other acute lower respiratory infections	J20-J22	3.63	4 December 2019	2.10	05 May 2021	−1.53 (−42.2)	2.83	25 October 2021	−0.8 (-22.02 %)
Other diseases of upper respiratory tract	J30-J39	11.03	28 February 2020	9.80	12 July 2021	−1.23 (−11.18)	11.30	24 January 2022	0.27 (2.42 %)
Other non-infectious diseases of the respiratory system	J40-J94	7.10	28 February 2020	7.27	15 July 2021	0.17 (2.35)	7.07	21 July 2021	−0.03 (-0.47 %)
Other diseases of the respiratory system	J96-J99	6.83	1 March 2020	6.80	19 October 2020	−0.03 (−0.49)	7.10	25 January 2022	0.27 (3.9 %)

The change in peak diagnosis rates for the during and postrestriction periods are relative to the prerestrictions period. For the during restrictions period, only peaks after 1 June 2020 were considered in order to allow the 2020 winter peak to subside.

ICD-10, International Statistical Classification of Diseases and Related Health Problems 10th Revision; RSV, respiratory syncytial virus.

**Figure 2:**
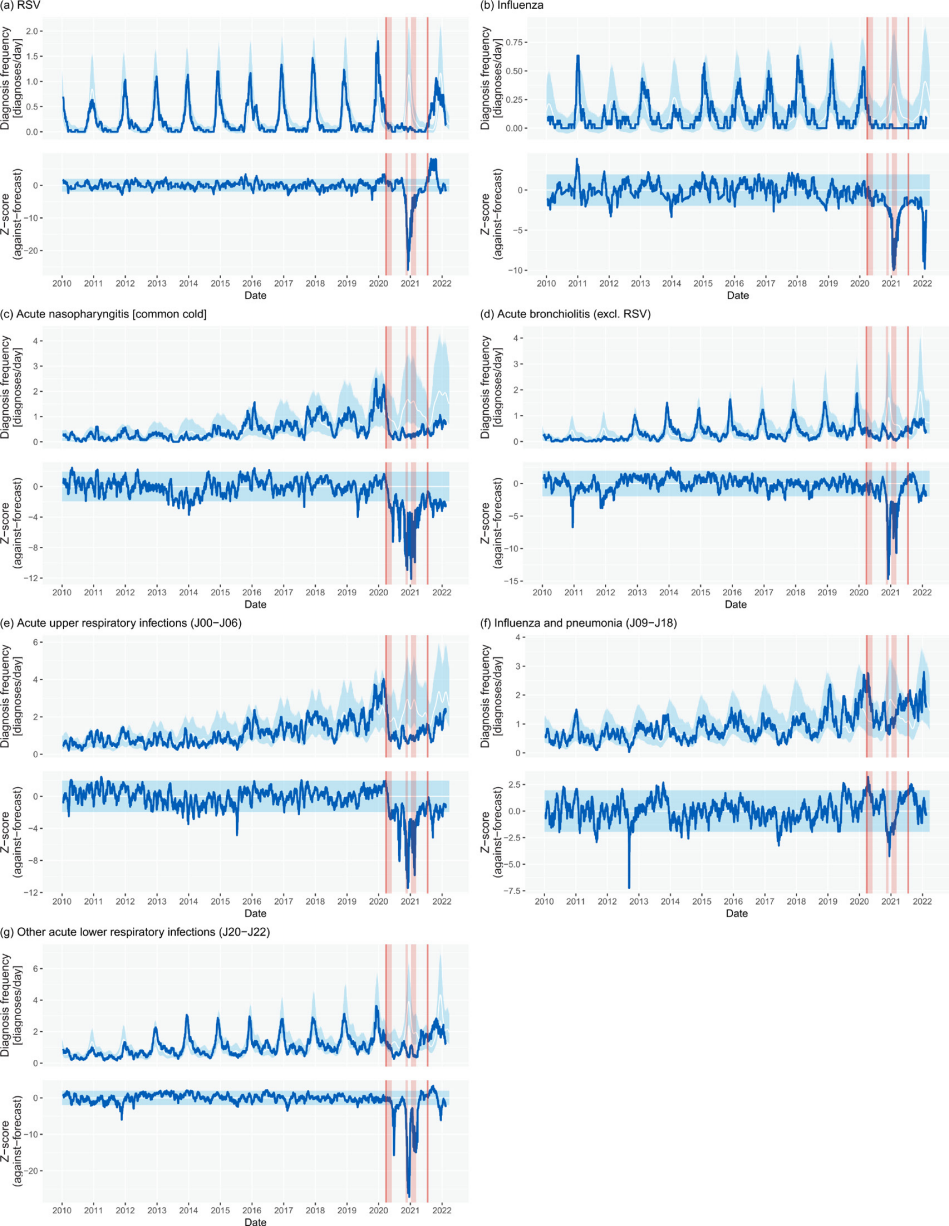
Diagnosis frequency forecast plots for the four seasonal respiratory disease categories: (A) RSV, (B) influenza, (C) acute nasopharyngitis and (D) acute bronchiolitis (excl. RSV), and three seasonal ICD-10 categories: (E) acute upper respiratory infections, (F) influenza and pneumonia and (G) other acute lower respiratory infections. In the diagnosis frequency plots, the blue line shows the observed 30-day moving average of daily diagnosis rate between 2010 and 2022. The white line shows the seasonal model forecast with the light blue 95% CIs. In the *z*-score plots, the blue line shows the observed diagnosis rate *z*-score against the forecast model. The light blue section shows the range for absolute *z*-score of less than 1.96 (95% CI). The vertical red lines define the start and end of widespread legal restrictions in response to the COVID-19 pandemic in England, UK. The light red sections show the three periods of national lockdowns. Specifically, note the marked reduction in rates for all respiratory infection groups during the pandemic restriction period but also the greater than expected rates for the period immediately postrestrictions relating to rising RSV infection rates. RSV, respiratory syncytial virus.

The Prophet seasonal model was calculated for all diagnosis categories based on the prerestriction period (figure 2, table 3). The seasonality amplitude of all four seasonal diagnosis categories were greater than 0.5, demonstrating notable seasonality. Additionally, three respiratory infection categories from the ICD-10 hierarchy (‘acute upper respiratory infections’, ‘influenza and pneumonia’, and ‘other acute lower respiratory infections’) were found to have seasonality amplitudes greater than 0.5. All categories had their seasonal peak identified between 26 November and 30 January annually (online supplemental table 2).

**Table 3:** Table of the forecast and observed number of diagnoses for each respiratory disease category in the during and postrestrictions periods

Diagnosis	ICD-10 codes	During restrictions (26 March 2020–18 July 2021)				Postrestrictions (19 July 2021–28 February 2022)			
		Forecast diagnoses (count)	Observed diagnoses (count)	Deviation from forecast diagnoses (count) (%)	Max/min *z*-score	Forecast diagnoses (count)	Observed diagnoses (count)	Deviation from forecast diagnoses (count) (%)	Max/min *z*-score
**Seasonal respiratory infection diagnosis categories**									
RSV	B974, J121, J205, J210	107.59	29	−78.59 (−73.05)	2.53/−25.93	94.67	120	25.33 (26.75 %)	8.13/−2.46
Influenza	J09, J10, J11	69.33	11	−58.33 (−84.13)	0.41/−9.96	47.35	7	−40.35 (-85.22 %)	−0.91/−9.81
Acute nasopharyngitis (common cold)	J00	538.98	161	−377.98 (−70.13)	0.8/−12.11	381.42	143	−238.42 (-62.51 %)	−0.62/−4.03
Acute bronchiolitis (excl. RSV)	J211, J218, J219	280.53	125	−155.53 (−55.44)	1.09/−14.67	209.96	138	−71.96 (-34.27 %)	1.66/−2.9
**ICD-10 hierarchy respiratory categories**									
Diseases of the respiratory system	J00-J99	9141.62	6803	−2338.62 (−25.58)	1.68/−12.88	6163.28	4284	−1879.28 (-30.49 %)	−0.48/−5.94
Acute upper respiratory infections	J00-J06	1027.44	496	−531.44 (−51.72)	1.53/−11.43	656.88	358	−298.88 (-45.5 %)	−0.11/−5.15
Influenza and pneumonia	J09-J18	590.69	678	87.31 (14.78)	3.22/−4.25	367.25	402	34.75 (9.46 %)	2.54/−1.22
Other acute lower respiratory infections	J20-J22	806.67	455	−351.67 (−43.6)	1.96/−27.19	553.03	442	−111.03 (-20.08 %)	3.3/−6.15
Other diseases of upper respiratory tract	J30-J39	3709.46	2851	−858.46 (−23.14)	1.7/−14.64	2251.77	1989	−262.77 (-11.67 %)	1.4/−1.36
Other non-infectious diseases of the respiratory system	J40-J94	2411.53	2432	20.47 (0.85)	2.2/−2.38	1512.69	1281	−231.69 (-15.32 %)	0.93/−2.15
Other diseases of the respiratory system	J96-J99	2461.88	2178	−283.88 (−11.53)	1.74/−3.06	1615.81	1188	−427.81 (-26.48 %)	0.35/−3.85

The deviation from forecast columns show the difference between the forecast and observed number of diagnoses for each period as well as the percentage deviation that this represents. The maximum and minimum *z*-scores for the observed moving average diagnosis rate relative to the forecast and CI are also given for both periods.

ICD-10, International Statistical Classification of Diseases and Related Health Problems 10th Revision; RSV, respiratory syncytial virus.

Comparing observed diagnosis to forecast diagnoses across the restriction period for the four seasonal diagnoses, all showed a greater than 50% reduction from expected rates. This included a 73%, 84%, 70% and 55% reduction for ‘RSV’, ‘influenza’, ‘acute nasopharyngitis’ and ‘acute bronchiolitis (excl. RSV)’, respectively. These categories also had significant negative minimum *z*-scores of less than −10.0 during the restrictions period.

Across the restrictions period, there was a general reduction of 26% in all ‘Diseases of the Respiratory System’ (J00–J99). Of the ICD-10 hierarchy categories considered in the study, all reduced against forecast rates except ‘Influenza and pneumonia’ (which contains pneumonia as the result of coronavirus infections) and the aggregated category ‘Other non-infectious diseases of the respiratory system’. All categories had negative minimum *z*-scores of less than −2.0 (outside the 95% CI); however, values were generally closer to zero than observed for the typically seasonal categories.

During the postrestriction period, there were large differences in diagnosis categories responses to the lifting of restrictions. Most categories have returned to, and remained, in-line with prerestriction forecasts; however some have not. RSV diagnosis rates rose most notably and were found to be consistently and significantly above the prerestrictions modelled forecast (maximum *z*-score 8.13), however subsequently returned to within forecast by the end of winter 2021/2022 (*z*-score <2.0). Additionally, both ‘influenza’ and ‘acute nasopharyngitis’ categories continue to show significantly reduced diagnosis rates in comparison with prerestrictions forecasts (*z*-scores −4.0 and −2.9 respectively).

## Discussion

In this study we have demonstrated, first, that mitigation and prevention measures put in place during the COVID-19 pandemic period were associated with significant reductions in the rates of children with a diagnosis of specific respiratory infections, particularly due to ‘RSV’, ‘influenza’, ‘acute nasopharyngitis’ and ‘acute bronchiolitis’, at a large children’s hospital in England, UK. Furthermore, the removal of prevention measures has resulted in widely varied responses in subsequent months. Second, we demonstrate the feasibility of applying an openly available machine learning forecasting model from another domain to routine electronic healthcare data within a secure digital hospital environment. Third, we use our method in analysing the seasonality of respiratory infections to showcase the potential of this model to clinical phenomena that are cyclical (eg, seasonal/diurnal). Our findings are consistent with known epidemiological data, suggesting robustness of the approach. Finally, the use of such a forecasting tool can identify unexpected deviations from normal, in this case the increasing rates of RSV infection in mid-late 2021 beyond the expected, allowing modelling of the likely peak in future months, hence aiding resource planning and public health measures. Again, the potential utility of this approach extends beyond the seasonality of respiratory infection alone.

### Clinical

The almost complete absence of the seasonal RSV infection pattern during the COVID-19 pandemic has been previously reported internationally,^4 7 20^ with larger than expected numbers susceptible postpandemic,^21^ and based on simulated trajectories from past data, significant RSV outbreaks had been predicted for the winter of 2021–22.^22 23^ Indeed, a resurgence of RSV infections above normal levels and at different times of the season has been reported in several countries.^24 25^ The data presented here confirm the significant reduction in RSV and other acute respiratory infections in London during the restriction period and further confirm greater than normal (predicted) rates occurring immediately following the lifting of restrictions. However, the peak diagnosis frequency rate was largely equal to that predicted for a ‘typical’ winter, based on our machine learning modelling, and by 28 February 2022 has returned to within the expected range. All other seasonal respiratory infections categories studied exhibited similar suppression in diagnoses during the restrictions period; however, (unlike RSV) they have all seen within or below forecast diagnosis rates postrestrictions. GOSH does not have an emergency department and is unique in relation to its patient population among children’s hospitals in the UK. Our absolute numbers of diagnoses for different respiratory infections including RSV are relatively low compared with district general hospitals, though the same seasonal and restrictions related effects have been widely observed.^4 7 26^ Despite this, the model was still able to forecast expected trends and deviations from previous years.

The results for diagnosis rate and number observed during winter 2021/2022, relative to forecast (particularly for RSV), are contrary to some of the previously published suggestions that a lack of population immunity due to the absence of cases during restrictions would lead to increased disease prevalence. Further study is required to explore if this finding is observed in larger, less selective populations as global restrictions are fully removed. However, if replicated elsewhere, these findings could imply that the risk of elevated infections and resulting disease is less of a risk for further increases in health service demand during periods where they are recovering from delays to a range of services during the pandemic.

### Machine learning modelling

The study illustrates the value of using routine healthcare data for secondary analyses within a bespoke data infrastructure based around well-defined data definitions and data models allowing data harmonisation, combined with the use of open and commonly used analytic tools such as R and Python,^17 27^ within a cloud-based trusted research environment allowing secure and auditable collaborative data analysis of non-identifiable data. This approach supports transferability to other organisations, and all code is available at https://github.com/goshdrive/seasonality-analysis-forecasting.

By applying a seasonal forecasting model^28^ to diagnosis data, we show how it is possible to generate forecasts with narrow confidence intervals from routine healthcare data, even when the underlying healthcare indicators are highly variable throughout a periodic cycle and/or involve moving year-on-year trends. By using a forecasting model that explicitly includes cyclical components described as a Fourier series, instead of a more generalised machine learning model, the library was able to tightly model the data with few parameters requiring domain-specific configuration. Specifically, these results were achieved by setting just three parameters specific to the indicators being studied. For this reason, the Prophet forecasting model has been successfully used in diverse areas including finance,^29^ temperature prediction,^30^ cloud computing resource requirements^31^ and predicting COVID-19 infection rates.^32 33^

## Conclusion

In conclusion, these data, based on routine EHR data combined with cross-domain time-series forecasting machine learning tools, demonstrate the near-complete absence of the seasonal acute respiratory infection-related diagnoses in a specialist children’s hospital during the period of the COVID-19 pandemic mitigation measures in 2020 and 2021. In addition, the data show an earlier-than-usual spike in RSV infection in 2021 but remained within the forecast range. The study illustrates the value of curated real-world healthcare data to rapidly address clinical issues in combination with the use of openly available machine learning tools, which can be applied to a range of scenarios relating to forecasting cyclical time series data.

## Data Availability

No data are available. No individual participant data will be available.
