# Supplementary material for: Machine learning forecasting for COVID-19 pandemic-associated effects on paediatric respiratory infections
Source: Arch Dis Child. 2022 Aug 10;107(12):e36. doi: 10.1136/archdischild-2022-323822 (PMC9685698; doi:10.1136/archdischild-2022-323822)
Supplement: Supplementary data [file archdischild-2022-323822supp001.pdf]

Supplemental Material

Supplemental 1: Table of cohort sizes for the diagnosis categories and three time periods. This shows the number of distinct diagnosis periods with a patient diagnosis in any of the ICD 10 codes shown.

Diagnosis	ICD-10 Codes	Total Observed Diagnoses [count]	Observed Diagnoses Pre Restrictions (01 January 2010 to 25 March 2020) [count]	Observed Diagnoses During Restrictions (26 March 2020 to 18 July 2021) [count]	Observed Diagnoses Post Restrictions (19 July 2021 to 28 February 2022) [count]
**Seasonal Respiratory Infection Diagnosis Categories**					
RSV	B974, J121, J205, J210	1,060	891	29	120
Influenza	J09, J10, J11	471	448	11	7
Acute nasopharyngitis [common cold]	J00	2,214	1,787	161	143
Acute bronchiolitis (excl. RSV)	J211, J218, J219	1,568	1,203	125	138
**ICD-10 Hierarchy Respiratory Categories**					
Diseases of the respiratory system	J00-J99	49,168	32,057	6,803	4,284
Acute upper respiratory infections	J00-J06	5,464	4,207	496	358
Influenza and pneumonia	J09-J18	4,688	3,071	678	402
Other acute lower respiratory infections	J20-J22	5,103	3,825	455	442
Other diseases of upper respiratory tract	J30-J39	19,906	12,515	2,851	1,989
Other non-infectious diseases of the respiratory system	J40-J94	16,739	10,876	2,432	1,281
Other diseases of the respiratory system	J96-J99	14,042	8,771	2,178	1,188

Supplemental 2: Table of the seasonality parameters calculated for the observed respiratory disease categories diagnoses in the pre restrictions period. The seasonality amplitudes have been calculated using the equation given in the methods section and the peak dates are given for categories exhibiting large seasonality with seasonality amplitudes greater than 0.5.

Diagnosis	ICD-10 Codes	Seasonality Amplitude [normalised]	Forecast Seasonal Peak
**Seasonal Respiratory Infection Diagnosis Categories**			
RSV	B974, J121, J205, J210	0.97	14 December
Influenza	J09, J10, J11	0.88	30 January
Acute nasopharyngitis [common cold]	J00	0.72	01 December
Acute bronchiolitis (excl. RSV)	J211, J218, J219	0.89	09 December
**ICD-10 Hierarchy Respiratory Categories**			
Diseases of the respiratory system	J00-J99	0.33	
Acute upper respiratory infections	J00-J06	0.55	26 November
Influenza and pneumonia	J09-J18	0.59	25 December
Other acute lower respiratory infections	J20-J22	0.76	13 December
Other diseases of upper respiratory tract	J30-J39	0.33	
Other non-infectious diseases of the respiratory system	J40-J94	0.31	
Other diseases of the respiratory system	J96-J99	0.31	

Bowyer SA, *et al. Arch Dis Child* 2022; 107:e1–e8. doi: 10.1136/archdischild-2022-323822
